# Barriers and Facilitators in the Admission of Underrepresented Groups to Medical School: A Scoping Review

**DOI:** 10.5334/pme.1931

**Published:** 2025-10-30

**Authors:** Oana Raluca Gröne, Mira Lou Stemmann, Josephine Nana Hill, Johanna Hissbach

**Affiliations:** 1 Selection Procedures Department, Institute of Biochemistry and Molecular Cell Biology, University Medical Center Hamburg-Eppendorf, Hamburg, Germany

## Abstract

**Introduction:**

Lack of diversity in the healthcare workforce harms patient care and outcomes, driving calls for more inclusive medical education. Physicians from disadvantaged backgrounds often serve underserved areas, and diversity improves cultural competence and trust. However, admissions still favour higher socioeconomic applicants, with barriers like standardized exams limiting access for underrepresented groups. This review examined barriers and enablers to medical school access for underrepresented groups, aiming to inform fairer admissions practices.

**Methods:**

A scoping review was conducted using Arksey and O’Malley framework to map literature on barriers and facilitators to medical school access for applicants from migration backgrounds and low socioeconomic status. A comprehensive search of PubMed, ERIC, and Google Scholar (Nov 2024–Apr 2025) included studies in five languages published in the past 10 years. Data were extracted in early 2025 and thematically analyzed using Braun and Clarke’s method.

**Results:**

Underrepresented groups face structural, institutional, economic, social and psychological barriers to medical school entry. Key challenges included financial hardship, inadequate academic support, lack of social capital, exclusionary institutional practices and psychological factors. However, targeted interventions – such as pipeline and outreach programs emphasizing mentorship and support networks – can help mitigate these barriers.

**Conclusion:**

Despite ongoing efforts to widen participation, underrepresented groups continued to face complex, intersecting barriers to medical school admission. Addressing these challenges required more than general policy initiatives – it called for intentional, community-based approaches tailored to students’ specific needs. This review highlighted the need for sustained, systemic change alongside targeted support strategies.

## Introduction

The lack of diversity among healthcare professionals has received growing attention in recent research due to its negative impact on healthcare interactions and health outcomes [1,2]. Additionally, workforce diversification has become a key health policy issue [3,4,5]. The underrepresentation of people from disadvantaged backgrounds in healthcare has broader implications for healthcare delivery. Research suggests that physicians from rural and low-income backgrounds are more likely to serve in underserved communities [6]. Addressing disparities in medical education could therefore contribute to a more equitable healthcare system. Additionally, a surge in diversity among medical professionals can enhance communication and patient care by fostering greater cultural competency and improving patient-provider trust [7].

Medical school admissions globally exhibit a strong socioeconomic bias, with students from wealthier backgrounds being significantly overrepresented. Studies from Australia [8], the UK [9,10], the USA [11,12], and Germany [13] highlight structural barriers that limit access for disadvantaged groups, including low-income, rural, and minority applicants. Nearly 80% of accepted medical applicants in the UK had parents in high-status professions [10], while 73.8% of medical students in Germany came from the highest socioeconomic quintile [13]. Socioeconomic status, parental occupation, ethnicity, and gender significantly influence admission outcomes [9,10,11,13]. This overrepresentation may reflect factors such as greater access to quality education, preparatory resources, and professional networks among wealthier students. Familiarity with the admissions process and financial stability can also provide advantages, while systemic biases may further reinforce disparities. These possible explanations highlight the complex interplay of social and structural influences shaping medical school admissions.

Despite differences between countries, studies consistently show that medical school admissions worldwide favour applicants with financial and social advantages, while disadvantaged groups face persistent barriers. Admission criteria – particularly standardized entrance exams such as the UK Clinical Aptitude Test (UKCAT) and the Graduate Medical School Admissions Test (GAMSAT) – tend to favour applicants from independent schools and wealthier backgrounds due to differences in educational opportunities and preparation resources [14,15,16]. However, although students from lower socioeconomic backgrounds tend to begin medical school with lower academic achievements, several studies show that they frequently close this gap over time and, in some instances, even surpass their more advantaged peers in subsequent academic performance [14,15]. This finding suggests that significant potential is being overlooked in the early stages of selection.

To address these disparities, various efforts have been made to widen participation of underrepresented groups in medical education. These efforts include alternative admission pathways and outreach initiatives, such as minority-serving institutions (MSIs) in the United States and equity pathways for Indigenous and rural students in New Zealand [17,18]. Additionally, admission procedures that emphasize intrinsic motivation, mentorship, and targeted recruitment efforts have also shown promise in increasing diversity [19,20]. However, the effectiveness of these interventions varies, and some scholars argue that systemic barriers in medical education persist, leaving underrepresented students to face ongoing obstacles despite widening participation efforts [21,22].

Understanding barriers and facilitators influencing the admission of underrepresented groups into medical school can help selection stakeholders take action in developing more equitable and holistic admission criteria. Rattani et al.’s systematic review examines the barriers and facilitators to pursuing careers in medicine for underrepresented groups, particularly Black students [23]. The review emphasizes the complex interplay of socioeconomic, psychosocial, and institutional factors in shaping access to medical education. Tackling barriers such as financial constraints, limited resources, and racism—while reinforcing facilitators like mentorship, psychosocial support, and revised admission criteria—can improve opportunities for these students to enter the medical field. However, the review primarily focuses on the American context, excluding European studies and non-English sources, and it includes, among others, non-empirical and non-peer-reviewed studies. Additionally, the authors’ definition of underrepresented groups is largely centred on Black applicants and consequently too narrow in a European educational context.

This literature review examines the factors that hinder or support access to medical school for underrepresented groups. By synthesizing findings from recent studies, this review aims to deepen the understanding of the factors influencing medical school admissions and to identify strategies for creating a more equitable and inclusive medical education system that better reflects the diversity of the populations it serves. The research question of our literature review is: What barriers and facilitators play a role in the admission of underrepresented groups to medical school?

## Methods

This scoping review follows the framework outlined by Arksey and O’Malley [24] which was further refined by Daudt et al. [25]. It employs systematic steps to identify and analyze literature on barriers and facilitators affecting the admission of underrepresented groups into medical school [26]. The scoping review method was chosen to systematically map the existing literature, allowing for the identification of key themes and research gaps. This approach is particularly suited for providing a broad yet structured synthesis of evidence in complex fields such as widening participation in medicine where the extent and nature of existing evidence are not yet well defined.

### Identifying the research question

The research question was developed as part of a larger project aimed at designing effective interventions to widen participation in medical schools. The question was formulated through collaborative discussions among the authors, reflecting their diverse expertise and backgrounds. ORG is a White female public health researcher with a migrant background and experience in health literacy research; MLS is a White female educational scientist and pedagogue; JNH is a Black female health scientist specializing in migrant health research; and JH is a White female psychologist with expertise in developing medical school admission selection procedures. This multidisciplinary team ensured that the research question addressed the complex factors influencing medical school admissions for underrepresented groups.

### Identifying relevant studies

A comprehensive literature search was performed using PubMed, ERIC, and Google Scholar. Search terms included combinations of barriers, facilitators, interventions, medical education, admissions, underrepresented groups (e.g., low socioeconomic status, migration background), and themes related to widening access, equity, and diversity. For this review, “underrepresented in medicine” is defined according to the Association of American Medical Colleges (AAMC) as “racial and ethnic populations that are underrepresented in the medical profession relative to their numbers in the general population” [27]. Additionally, “representativeness of the student body” refers to the extent to which medical students reflect the diversity of the broader population or specific target groups. Our review focuses on two key dimensions of diversity: ethnic groups/minorities (in international studies) or individuals with a migration background (in German studies) and low socioeconomic status, including First-in-Family (First-Generation) students. A detailed description of our search is presented in appendix 1.

### Selecting studies

Inclusion and exclusion criteria ensured the systematic identification of studies relevant to the research objectives, enhancing the transparency, consistency, and reproducibility of the scoping review while minimizing potential bias. To be included in this review, studies had to explicitly examine perceived barriers and facilitators in the medical school selection process, focus on participants that belonged to underrepresented groups, as defined above, be published within the past 10 years and be available in English, German, Spanish, French, or Romanian. This timeframe was chosen to ensure that the review reflects the most current evidence and developments in the field, particularly given the evolving nature of widening participation policies and selection procedures for medical schools. The publication languages were selected because they are the primary languages of the research team, ensuring accurate interpretation and analysis of the content. Moreover, we tried to minimize language bias by including studies in more than one language (English) and searching databases in multiple languages. We excluded studies focusing on transition into specialty training rather than medical school admissions and non-peer-reviewed articles such as editorials, book chapters, opinion pieces, or commentaries.

### Charting and summarising the data

**Figure 1:**
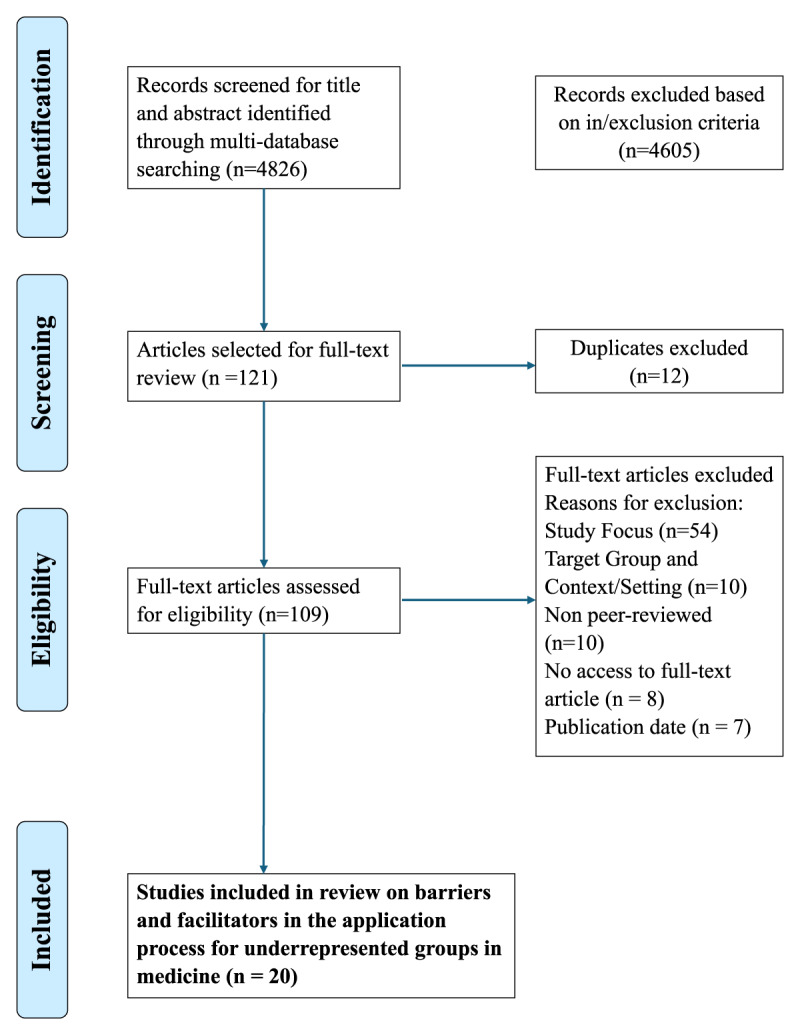
Flow diagram (Prisma) for the scoping review about barriers and facilitators to the admission of underrepresented groups to medical school.

A three-stage screening process ensured consistency among authors. After removing duplicates, three authors (ORG, MLS and JNH) reviewed titles and abstracts based on inclusion criteria, resolving disagreements through discussion. Full texts were then independently assessed, with discrepancies again discussed. Key data from included studies—such as study details, populations, and identified barriers and facilitators—were extracted and compiled in Excel (Appendix 2). A PRISMA flow diagram (Figure 1) illustrates the selection process.

Thematic analysis, following Braun and Clarke’s approach, was used to identify patterns related to barriers and facilitators in medical school admissions [28]. The first author coded data manually and developed initial themes, which were then reviewed and refined collaboratively with all co-authors to ensure clarity and accuracy. We conducted a thematic analysis to organize and describe key findings across the included studies. In line with established scoping review methodologies, this analysis was undertaken in a descriptive manner, rather than aiming for deep interpretive insights. The goal was to identify and group common patterns, concepts, and categories to map the breadth and nature of the available literature. This approach supports the transparent presentation of results and facilitates an understanding of the scope and distribution of evidence on the topic.

## Results

### Characteristics of included studies

Our search resulted in 4826 article titles, of which 121 articles were chosen for full-text screening. After removing 12 duplicates, 109 articles remained for further review. After applying our inclusion criteria, 20 articles were included in the final review.

All included studies were published in English, and, with one exception [29], originated from English speaking countries. Regarding the country of origin of studies, ten were from the UK [30,31,32,33,34,35,36,37,38,39], five from the USA [40,41,42,43,44], three from Australia [45,46,47], one from Canada [48] and one from the Netherlands [29] (Table 1).

**Table 1:** Characteristics of articles identified in the scoping review.


DOMAIN	FEATURE	NO OF ARTICLES (N = 20)

**Country**	Australia	3

	Canada	1

	The Netherlands	1

	UK	10

	USA	5

**Year of publication**	2016–2020	9

	2021–2025	11

**Study design**	Qualitative	18

	Quantitative	1

	Mixed-methods	1

**Characteristic of underrepresented groups**	Rural background	1

	First generation students	1

	Ethnicity	4

	Socio-economic status	2

	Not specified or defined as non-traditional	12


All but two studies [36,46] used a qualitative methodology, mainly focusing on perceptions of barriers and facilitators to the admission of underrepresented groups into medical school.

### Thematic analysis of included studies

The reviewed studies highlight that admission to medical school is highly competitive, posing significant challenges—especially for students from underrepresented and disadvantaged backgrounds. However, several facilitators help mitigate these barriers and support applicants in navigating the admissions process. As outlined in Table 2, barriers and facilitators to admission to medical school include three main domains: structural and institutional, social and economic, as well as motivational and psychological factors.

**Table 2:** Main themes identified in included studies.


BARRIERS	NO OF ARTICLES (N = 20)	FACILITATORS	NO OF ARTICLES (N = 20)

**Structural and institutional factors**			

Under-resourced and underperforming schools	7	Teachers and peer support	5

Lack of sensitivity for diversity	1	Positive institutional practices and supportive culture (assignment of tutors, community partnerships, use of inclusive language, contextualised admissions, participation of underrepresented groups in selection committees)	5

Complex application and selection process, academic requirements, lack of clear information	11	Pipeline and outreach programs (mentorship, shadowing and internship opportunities, accessible information, resources and guidance)	10

**Economic and social factors**			

Financial challenges	10		

Lack of social capital (personal connections)	6	Social networks and resources	7

Family pressure, no emotional support	4	Family support and encouragement	5

**Motivational and psychological factors**			

Lack of motivation, low expectations from teachers, concerns about health system and job prospects, scepticism regarding new medical schools	7	Genuine interest in medicine, desire to help others, role models	5

Low self-confidence, imposter syndrome, feelings of alienation	7	Trust in their own abilities, resilience	4


#### Structural & institutional factors

Due to disparities in educational quality, students in **underfunded schools** may struggle to meet the academic and extracurricular requirements for medical school admission [32]. Underfunded schools are defined here as educational institutions that lack adequate financial resources to provide essential services, materials, and support for effective teaching and learning. These schools frequently offer limited support for the application process, including insufficient preparation for medical school application and admission [34,36,47]. Many students lack access to formal career guidance and mentoring, which limits their ability to make informed decisions about pursuing medicine [32,42]. Additionally, **limited resources** in schools—such as reduced teaching hours [31], insufficient support for entrance exams [38], and staff shortages [31]— compound these challenges. For example, while guidance teachers had some designated time to discuss career choices, it was typically limited to brief annual meetings, providing minimal opportunity for in-depth advice or support for students aspiring to a medical career [31].

**Support from teachers and peers** played an essential role in fostering student confidence and readiness for medical school applications. Teachers provided practical assistance—such as mock interviews and personal statement guidance—while also encouraging self-reflection to ensure medicine aligned with students’ interests [31]. Peer support with the application process [34] and peer networks organized at the institutional level further provided vital support, allowing students to share challenges, practice interviews, and encourage each other in the process [31,42,44,45].

A **lack of sensitivity for diversity** in medical schools persists, as universities often favour applicants who fit traditional academic profiles and uphold narrow definitions of merit [33]. Students from underrepresented backgrounds frequently experience marginalization, as their perspectives and challenges are often overlooked in institutional decision-making [33]. The use of distancing language and stereotypes in academic institutions that frame underrepresented students (often referred to as widening access or widening participation students) as socially and academically inferior, along with lower expectations of their competence and aspirations reinforce barriers in the admission process [33].

The **application and selection process** itself can be daunting, involving multiple forms, aptitude tests, and interviews that may be unfamiliar to applicants from underrepresented backgrounds [39,47]. Moreover, while widening participation initiatives aim to improve access, they sometimes introduce additional administrative burdens, making the application process more complex for students from underrepresented groups [39]. Strict **academic requirements** and the competitiveness of medical school admissions discourage many students and create another major barrier, with many students perceiving the high entry standards as unattainable [30,31,34,42]. The strong emphasis on academic achievement over personal attributes further excludes talented candidates who may not have had access to top-tier education [34]. Finally, a **lack of clear, accessible information** about medical careers, entrance exams, and selection criteria [29,34,39,41,42] — combined with the absence of knowledgeable family support [34,36,38,40], particularly for underrepresented and first-generation students — makes navigating the complex medical school application process especially challenging.

**Positive institutional strategies and supportive culture** played a significant role in reducing barriers for prospective medical students. The **assignment of personal tutors** offered students guidance and reassurance during the application process, while **community partnerships**, such as with local healthcare trusts, offered valuable real-world experience through internships [32]. Institutions that adopted **inclusive and affirming language**, such as referring to widening participation students as “our students,” helped create a respectful and integrated learning environment where all students felt valued and part of the medical community [33]. Rural graduates viewed **contextualised admissions**, including equity bonuses for rural, experienced, or financially disadvantaged applicants, as effective tools for widening access [47]. Increasing diversity in admission panels, including **participation of underrepresented groups in interviews assessment and selection committees**, contributed to fairer evaluations and reduced bias [48]. Furthermore, a culture that values diversity and inclusion was seen as essential, with study participants highlighting the importance of diverse role models in reinforcing a sense of belonging in medicine [30].

Structured **outreach and pipeline programs** have been instrumental in increasing access to medical careers by building students’ skills, confidence, and understanding of the admissions process. Pipeline initiatives offering exam prep and long-term support were especially effective for first-generation and underrepresented students [40]. **Mentorship**, particularly from mentors with similar backgrounds, provided both guidance and emotional support, fostering a sense of belonging [35,38,40,41]. **University outreach** efforts, including school partnerships and campus visits, helped demystify the application process through targeted support like tutoring [31,34,36,38]. Some newer medical schools also prioritized local, less affluent populations, offering tailored resources and stronger community engagement [32]. Finally, clinical exposure through **shadowing and internships** reinforced students’ motivation, clarified career goals, and strengthened applications, especially when access was facilitated by mentors or medical schools to support disadvantaged students [34,40,44].

Often mentioned in the literature as facilitating admission into medical school was **access to clear, accurate information** about medical school requirements, which was vital in building student confidence and readiness. Step-by-step **guidance** on admissions tests, personal statements, and interviews helped students better understand the process [29,40]. Printed and online **resources** clarified key details such as deadlines, financial aid, and application expectations [34]. Additionally, online communities and social media groups offered ongoing peer support and advice [34,35].

#### Economic and social factors

**Financial challenges** were among the most frequently cited obstacles in the included studies. The high costs associated with applying to medical school — including tuition fees, entrance exams, living expenses and interviews (with travel and secondary application fees being particularly common in the United States) — pose significant hurdles, particularly for those from lower socioeconomic backgrounds [29,32,36,40,42,45,46,47]. Many students must work alongside their studies to afford these costs, which can negatively impact academic performance and preparation [38,40,47]. Additionally, lack of accessible information about financial aid options [32] combined with limited financial literacy [41] deters applicants and further reinforces the perception that medical education is unattainable.

Beyond financial barriers, **social capital** plays a critical role in shaping access to medicine. Students from underrepresented backgrounds often lack **personal connections** to medical professionals, limiting their access to shadowing or clinical experience opportunities [29,34,35,42,45,47]. In contrast, students with family members in healthcare or with a good social network benefit from early exposure, work experience, mentorship, and valuable guidance that can strengthen their applications and clarify career goals [29,30,34,36,38,42,44].

**Family support** also emerged as a key factor influencing students’ pathways into medicine. **Encouragement** from family members, especially for first-generation college students, is a powerful motivator and source of resilience after setbacks like rejection or poor test scores [36,38,40,41]. In some cases, families played an active role by participating in on-campus events and fostering accountability for academic success [44]. However, family expectations could be a double-edged sword — while motivating for some students, others felt pressured to pursue medicine regardless of personal interest, leading to unrealistic goals or diminished motivation [30,31]. Conversely, the absence of emotional support from family members is a significant barrier to applying to and succeeding in medical school [38,42].

#### Motivational and psychological factors

Societal perceptions of medicine as a prestigious and high-status profession often foster extrinsic motivations among applicants, such as the pursuit of financial stability and social recognition [30,31]. However, among underrepresented groups, **a general lack of motivation** has been observed [29], often exacerbated by **low expectations from teachers**. This discouragement can significantly influence students’ career choices, reinforcing educational inequalities and reducing the likelihood of pursuing medicine [31,33,34,41]. Broader **concerns about the healthcare system and uncertain job prospects** may further diminish students’ motivation and interest in a medical career [34,42]. Additionally, **scepticism** surrounding the credibility of newer medical schools—often viewed as less established within traditional academic hierarchies—can create additional hesitation [32].

Psychological barriers such as **low self-confidence** and **imposter syndrome** also play a critical role in deterring students, particularly those from disadvantaged backgrounds. Many prospective applicants fear failure or feel they do not belong in the competitive environment of medical education [29,31,34,40,47]. Students from underrepresented backgrounds also frequently report **feelings of alienation** and difficulty integrating into the culture of medical school [45]. These experiences are often intensified by stereotypes that question their academic competence and ambition, contributing to a sense of exclusion and discouraging them from applying [33]. Negative or discouraging interactions with teachers and advisors, combined with insufficient support, further undermine students’ confidence and aspirations [34,40]. Finally, concerns about the length, intensity, and emotional demands of medical training frequently amplify doubts and hesitation among prospective applicants from underrepresented backgrounds [29].

Motivational and psychological factors were also found to facilitate the admission of underrepresented groups into medical programs as students demonstrated strong intrinsic motivation and personal drive. A genuine **interest in medicine**, fascination with the human body, and a desire to help others were commonly cited as key motivators [29,30,42]. For some, the goal of addressing healthcare inequities and contributing to social justice was a powerful incentive [32]. Students expressed **trust in their own abilities** [38] and many demonstrated **resilience** and determination to persist in the face of academic, financial, or social challenges [40,47]. In fact, some viewed the competitiveness of medical school admissions not as a deterrent but as a motivating challenge — an opportunity to prove their capabilities [29]. Lastly, seeing **role models** from similar backgrounds, particularly underrepresented minority physicians, reinforced the belief that a medical career was both attainable and worthwhile [29,43].

## Discussion

The current review emphasizes that access to medical education remains shaped by structural, institutional, and social inequalities that disadvantage students from underrepresented backgrounds. Barriers such as limited academic preparation, financial constraints, lack of social capital, and insufficient guidance compound the challenges of navigating a complex and competitive admissions process. These factors are often reinforced by exclusionary institutional practices and stereotypes, which undermine applicants’ confidence and sense of belonging. Crucially, these barriers are not discrete but deeply interrelated. Drawing on the concept of intersectionality [49], the findings suggest that structural, economic, psychological, and social dimensions of disadvantage intersect in complex and compounding ways that resist simple categorisation. For instance, financial barriers and socioeconomic background often correlate with reduced access to mentoring and academic support, while institutional biases may exacerbate experiences of marginalisation and imposter syndrome.

At the same time, the studies included in our review literature highlight that targeted interventions — including pipeline and outreach programs, mentorship, inclusive admissions practices, and exposure to medical environments through internship and shadowing — can play a significant role in reducing these disparities. Support from family, teachers, and peers, alongside strong intrinsic motivation and resilient self-belief, further empowers underrepresented students to overcome structural disadvantages and access medical education.

Results of our literature review resonate with those reported in the earlier review conducted by Rattani et al. [23]. While both reviews address barriers and facilitators within medical education, our review offers a broader global perspective on underrepresented groups, whereas Rattani et al.’s review provides a focused analysis on the unique challenges faced by Black premedical students in the USA. However, both reviews reveal overlapping themes such as socioeconomic burdens, lack of access to preparatory materials and academic enrichment programs, inadequate exposure to the medical field, and poor mentorship [23]. This suggests that certain barriers are universal across different underrepresented populations and contexts, reinforcing the idea that structural inequities are deeply embedded in the medical education pipeline.

We explored how barriers to medical education compare to those faced by prospective students in other health-related professions. A recent scoping review by Mohammed and Roberts (2024), which examined perceived barriers among underrepresented minority students applying to health science programs, revealed notable parallels with our findings [50]. At the individual level, students frequently reported limited understanding of the application process, self-doubt, and concerns about family separation [50]. Socio-structural barriers such as lack of familial support, fear of discrimination and the absence of mentorship were also common [50]. Additional studies examining the underrepresentation of Latin American and African American students in fields like nursing, pharmacy, and public health highlighted further obstacles, including poor academic performance in science subjects, insufficient early educational preparation, and significant financial constraints [51,52]. These findings indicate that underrepresented students in various health professions often face shared structural and socioeconomic challenges, although the severity and scope of these barriers may differ across fields.

The findings of our review underscore how institutional, social, and economic barriers shape access to medical education, though the extent and nature of these barriers may vary across different educational contexts. In Germany, for instance, most universities are publicly funded, which theoretically lowers financial barriers. Yet students from socially disadvantaged backgrounds and families with lower educational attainment are underrepresented in medicine [13]. These students often face hurdles such as lower Abitur (the German school-leaving qualification, broadly comparable to A levels in the UK) grades [53], which reduce their admission chances, compounded by the relatively small proportion of disadvantaged individuals who complete the Abitur at all [54]. Similarly, as the review shows, social capital, financial stability, and access to preparatory resources are critical facilitators worldwide. In the USA and UK, high tuition fees, the reliance on standardized entrance exams, and unequal access to mentoring and preparatory courses mirror the same inequalities identified in this review. While targeted interventions—such as widening participation schemes, scholarships, and alternative pathways—have been introduced to improve access, both the German and Anglo-American systems continue to reflect entrenched social inequities that hinder equal opportunity in medical careers.

### Strengths and limitations

A key strength of our review is its exclusive focus on empirical studies, ensuring that the findings are grounded in systematically collected data rather than theoretical or anecdotal accounts. By organizing the findings into thematic categories — structural and institutional, social, economic, motivational, and psychological — the review provides a nuanced understanding of the multifaceted nature of access to medical education. However, the review may reflect geographic or cultural biases, especially since the majority of the research originates from specific countries (UK, US, the Netherlands, and Australia). The inclusion of peer-reviewed articles also risks underrepresenting unpublished program evaluations or local initiatives in the grey literature that might offer practical insights. Furthermore, the review largely draws on qualitative and two observational studies, which, while rich in context, can limit generalizability.

### Implications for policy, practice, and research

The findings of this review suggest several practical steps to address the persistent inequalities in access to medical education. Medical schools should implement contextualised admissions policies that evaluate applicants not only on academic performance but also in light of their social, economic, and educational circumstances. Taking into account factors such as school quality, socioeconomic status, and lived experience would enable a more equitable and inclusive admissions process. Medical schools should expand pipeline and outreach programs with a focus on mentorship and early exposure, particularly for students and families from underrepresented backgrounds. Partnerships with schools and communities can foster inclusion, while financial support and alternative admissions routes (e.g., vocational pathways or preparatory programs) can help address financial and social barriers. Institutions must also address internal biases by training admissions and academic staff in diversity and inclusion. Promoting diverse representation on admissions panels can help challenge existing stereotypes and improve equity.

Future research should prioritise longitudinal studies to assess the long-term impact of widening participation efforts and alternative admissions. Cross-country comparisons (e.g., Germany and the Netherlands) could help identify effective strategies, while interdisciplinary research could offer new insights into addressing persistent inequalities.

## Conclusion

This review highlights that access to medical education is profoundly influenced by structural, institutional, and social inequalities that continue to disadvantage underrepresented students. While barriers such as financial hardship, limited academic support, and lack of social capital persist, the evidence also points to the transformative potential of targeted interventions and personal resilience. By addressing systemic exclusion and investing in inclusive strategies, medical education systems can move toward greater equity and ensure that talent is not lost to circumstance. This is not only vital for fairness in education, but also essential for improving healthcare outcomes, as a more diverse medical workforce enhances cultural competence, reduces health disparities, and strengthens patient trust across communities.

## Additional File

The additional file for this article can be found as follows:

10.5334/pme.1931.s1 Appendices. Appendix 1 and Appendix 2.
