# Supplementary material for: Barriers and Facilitators in the Admission of Underrepresented Groups to Medical School: A Scoping Review
Source: Perspect Med Educ. 2025 Oct 30;14(1):673–84. doi: 10.5334/pme.1931 (PMC12577556; doi:10.5334/pme.1931)
Supplement: Appendices. — Appendix 1 and Appendix 2. [file pme-14-1-1931-s1.pdf]

Appendix 1: Search strategy and results

Database	Search Term & Filter	Results	Selected
PubMed	barrier* AND (admission OR selection) AND medical school* Filters: in the last 10 years, English, German, Humans, Adult: 19+ years	1453	10
PubMed	barrier* AND selection AND medical school* Filters applied: in the last 10 years, English, French, German, Romanian, Spanish, Humans, Adolescent: 13-18 years, Child: birth-18 years, Adult: 19+ years, Young Adult: 19-24 years	1413	3
PubMed	(migration background OR underrepresented group OR underrepresented in medicine) AND (medical school OR admission OR medical education) AND (barrier OR facilitator) Filters: in the last 10 years, English, French, German, Romanian, Spanish, Humans, Child: birth-18 years, Adolescent: 13-18 years, Adult: 19+ years, Young Adult: 19-24 years	336	21
ERIC	Access AND medicine; filter: access to education (descriptor)	58	24
PubMed	("widening participation" OR "widening access") AND (medical school OR medical education) Filters: in the last 10 years, English, French, German, Romanian, Spanish, Humans, Child: birth-18 years, Adolescent: 13-18 years, Adult: 19+ years, Young Adult: 19-24 years	38	20
PubMed	"Students, Medical"[MAJR] AND (selection OR admission) AND barrier* Filters: in the last 10 years, English, French, German, Romanian, Spanish, Humans, Child: birth-18 years, Adolescent: 13-18 years, Adult: 19+ years, Young Adult: 19-24 years	25	3
PubMed	Admission AND medical school<br>Filter: 2014-2024, German	18	7
Google Scholar	Barrieren AND Zulassung AND Medizinstudium AND (soziale Benachteiligung OR sozioökonomische Benachteiligung)	83	1
Google Scholar	Zugang* "Medizinstudium" AND (soziale Benachteiligung OR sozioökonomische Benachteiligung)<br>Filter: 2014-2024, German	608	3
Google Scholar	Barriers* AND Facilitators* AND "Medical School" AND Admission*<br>Filter: 2014-2024, German	30	1
Google Scholar	widening access* AND "Medical School" AND Admission*	339	3

	Filter: 2014-2024, German		
Forschungsdaten Bildung ()	Studium Medizin Zulassung	247	4
Google Scholar	barrier* AND (admission OR selection) AND medical school*<br>Filters: 2014-2024, English	122	5
Google Scholar	barrier* AND selection AND medical school*<br>Filters: English, 2014-2024	33	1
Google Scholar	Barriers* AND Facilitators* AND "Medical School" AND Admission*<br>Filters: English, 2014-2024	3	2
Google Scholar	widening access* AND "Medical School" AND Admission*<br>Filter: English, 2014-2024	19	13

Appendix 2: Detailed information on study characteristics

**Author, year of publication, title**	**Country, setting**	**Study design and year of data collection**	**Participant characteristics**	**Objective of the study**
Alexander et al. 2019. Bridging the cultural divide? Exploring school pupils´ perceptions of medicine	UK, high schools	Qualitative, focus groups, October and November 2016	71 high-achieving school pupils (aged 16-18), primarily female (69.1%), ethnically diverse, from non-traditional backgrounds	To explore the perceptions of school pupils from non-traditional backgrounds regarding the barriers and motivations for pursuing a career in medicine and to evaluate whether the perspectives on access to medicine have changed compared to previous studies.
Alexander et al. 2021 “It’s going to be hard you know…” Teachers’ perceived role in widening access to medicine	UK, high schools and sixth-form colleges	Qualitative, interviews, 2019 to 2020	11 teachers from various school types (e.g., high schools, sixth-form colleges) with diverse experiences in educational counselling, spanning different geographical regions	To explore and understand teachers' perspectives and practices regarding how they support or hinder their students' aspirations for a career in medicine
Beattie et al. 2025 “I Hadn't Ever Really Thought It Was Something That I Could Do’: Rural Background Medical Graduates' Pathways to Medicine	Australia, rural longitudinal integrated clerkship	Qualitative, interviews, February-November 2022	39 integrated clerkship graduates, including 17 with a rural background before entering medicine (mean age 34)	To elevate the voices of rural background doctors by exploring their experience of applying for medicine and identifying learnings that can further widen access for perspective rural applicants
Brosnan et al. 2016 Experiences of medical students who are first in family to attend university	Australia, university	Qualitative, interviews, 2014	22 first-in-family medical students, including individuals from all five year levels of the medical program (77% female, average 24 years), mostly with socioeconomically disadvantaged backgrounds	To identify barriers faced by first-in-family students in medical school.
Burns et al. 2021 Assessing the pipeline: Perceived barriers to applying to dental school among pipeline program alumni	USA, University College of Dentistry	Qualitative, focus groups, 2020	23 alumni from NYU College of Dentistry's pipeline programs (aimed at underrepresented minorities and low-income students), average age 22 years, 78% first-generation college students	To understand the perceived barriers that underrepresented minority students experience when applying to dental school.
Cleland et al. 2018 Aspirations of people who come from state education are different": How language reflects social exclusion in medical education	UK, medical schools	Qualitative, interviews, 2018	Admissions Deans and staff from 24 UK medical schools	To examine the language used by Admissions Deans in representing widening access (WA) and under-represented minority (URM) students, focusing on how language reflects social exclusion and existing institutional values concerning diversity.

Cleland et al. 2024 Translating government policy into practice: How new UK medical schools enact widening participation	UK, medical schools	Qualitative, interviews, November 2023 - March 2024	6 Deans and 14 admissions staff from 6 newly established medical schools in England.	To examine how the policy of widening participation (WP) is enacted in six new medical schools and what challenges and successes arise in this process.
Dixon et al. 2020 Factors that Influence Underrepresented in Medicine (UIM) Medical Students to Pursue a Career in Academic Pediatrics	USA, medical schools	Qualitative, focus groups, 2016	20 UIM medical students	To explore the experiences of medical students who are underrepresented in medicine (URiM) and interested in pursuing academic pediatrics, as well as to identify supportive factors and barriers.
Eguiguren Wray et al. 2024 An Investigation into the Contextual Admissions Information Available at UK Medical Schools' Websites: What Are the Opportunities for Enhancement?	UK, medical school	Qualitative, 2020	57 undergraduate medicine courses at UK universities for the 2020 entry	To investigate the clarity and accessibility of contextual admissions information presented on UK undergraduate medicine course websites from the view of prospective students.
Freeman et al. 2016 Understanding the Leaky Pipeline: Perceived Barriers to Pursuing a Career in Medicine or Dentistry Among Underrepresented-in-Medicine Undergraduate Students	USA, colleges and universities	Qualitative, September 2012 - February 2013	82 students from 11 colleges, the majority were underrepresented in Medicine (61% Black/African American, 31% Latino/Hispanic, and other ethnic groups), median age 21.4 years	To examine the perceptions of barriers to pursuing a career in medicine or dentistry among URiM students and identify potential intervention areas to improve diversity in medical and dental schools.
Kumar et al. 2018 Testing for medical school selection: What are prospective doctors' experiences and perceptions of the GAMSAT and what are the consequences of testing?	Australia, medical school	Mixed-methods study, unclear data collection date	447 participants, 46% recruited from graduate entry medical school and 54% from an online GAMSAT preparation forum	To investigate how current medical school students and applicants experience and perceive the GAMSAT (graduate medical school admission test) and the consequences of testing
Martin et al. 2018 Widening interest, widening participation: factors influencing school students' aspirations to study	UK, medical school	Action research (nominal group technique), 2015	122 Year 12 (age 16–17) and 170 Year 9 (age 13–14) school children who participated in a widening participation program	To identify barriers or deterrents perceived by prospective medical students in applying to medical school, and examine strategies to address these obstacles, including the optimal stage in their academic careers for intervention and the agencies responsible for

medicine				implementing these solutions.
Melro et al. 2023 Front row seat: The role MMI assessors play in widening access to medical school	Canada, medical school	Qualitative, interviews, 2021	10 MMI assessors	To investigate whether MMI assessors carry unique insights on widening access (WA) to medical school.
Mulder et al. 2022 Influence of social networks in healthcare on preparation for selection procedures of health professions education: a Dutch interview study	The Netherlands, secondary school	Qualitative, interviews, 2019-2020	26 high school students from 14 schools in 5 cities in NL with traditional and non-traditional backgrounds interested in health professions studies (medicine among others)	To understand the perceptions of traditional and non-traditional students regarding facilitators and barriers in preparing for health professions education selection procedures, and to determine the role of social networks in their decision-making and preparations to apply.
Nicholson and Cleland 2017"It's Making Contacts": Notions of Social Capital and Implications for Widening Access to Medical Education	UK, medical school	Qualitative, focus groups and interviews, 2011-2015	Study A: 19 senior secondary (high) school students and/or foundation students on widening access programmes<br>Study B: 14 first year medical students from two urban medical schools in Scotland<br>Study C: focus groups and 15 follow-up individual interviews) study with senior (clinical years) medical students	To explore the notion of social capital at a micro (individual) level, how it is created, the different forms it takes, and how it works in relation to opening up new opportunities for medical applicants and students from lower socio-economic groups.
Sartania et al. 2021 Barriers to access, transition and progression of Widening Participation students in UK Medical Schools: The students' perspective	UK, medical school	Observational, unclear data collection date	125 medical students from three medical schools (with widening participation "flags") who had participated in widening access programs	To explore the nature of the barriers - both real and perceived - to accessing, transitioning and progressing in the medical education system.
Staley et al. 2023 Widening participation in medicine: A focus group evaluation of mock multiple mini-interviews for medical school applicants	UK, medical school	Qualitative, focus groups, 2022	28 participants, year 12 pupils	To evaluate the effectiveness of mock multiple mini interview (MMI), designed using guidelines created by the Medical Schools Council.
Ulloa et al. 2018 Perceptual and Structural Facilitators and Barriers to Becoming a	USA, medical school	Qualitative, interviews,	23 surgeons	To investigate structural and perceptual barriers that contribute to an underrepresentation of African-American and Latino surgeons.

Surgeon: A Qualitative Study of African-American and Latino Surgeons				
Weiss et al. 2024 Perspectives on Medical School Admission for Black Students Among Premedical Advisers at Historically Black Colleges and Universities	USA, medical school	Qualitative, focus group, interviews, 2019-2021	26 premedical advisers from historically black colleges and universities (4 self-identified as Asian, 19 Black or African American, 1 as Hispanic or Latino, and 2 as White). Thirteen participants had a doctorate degree, and 8 had more than 10 years of experience as a premedical adviser	To examine barriers to and facilitators of medical school matriculation.
Wilson and Hunter 2023 Access and Emergence: A Case Study of a Working-Class Journey into Studying Medicine	UK, secondary school	Qualitative, case study, 2012-2015	10 pupils from a Scottish secondary school who were being mentored (by retired middle -class professionals) and who were applying to more competitive courses in higher education	To assess to what extent institutional practices advance or impede access to higher education with a focus on medicine.
